# Supplementary material for: Efficacy and Safety of L-Menthol During Gastrointestinal Endoscopy—A Systematic Review and Meta-Analysis of Randomized Clinical Trials
Source: J Clin Med. 2025 Jun 17;14(12):4296. doi: 10.3390/jcm14124296 (PMC12194438; doi:10.3390/jcm14124296)
Supplement: Supplementary file 1 [file jcm-14-04296-s001.zip › jcm-3678440-supplementary.pdf]

Supplementary Material

Efficacy and safety of L-Menthol during gastrointestinal endoscopy –

A systematic review and meta-analysis of randomized clinical trials

Authors

Dorottya Gergő[1,2], Andrea Tóth-Mészáros[2], Alexander Schulze Wenning[2], Péter Fehérvári[2,3], Uyen Nguyen Do To[2,4], Péter Hegyi[2,5,6], Bálint Erőss[2,5,6], Attila Ványolós[1,2,7] and Dezső Csupor[2,5,8]*

Affiliations:

1 Department of Pharmacognosy, Semmelweis University, 1085 Budapest, Hungary

2 Centre for Translational Medicine, Semmelweis University, 1085 Budapest, Hungary

3 Department of Biostatistics, University of Veterinary Medicine, 1078 Budapest, Hungary

4 András Pető Faculty, Semmelweis University, 1125 Budapest, Hungary

5 Institute for Translational Medicine, Medical School, University of Pécs, 7624 Pécs, Hungary

6 Institute of Pancreatic Diseases, Semmelweis University, 1083 Budapest, Hungary

7 Center for Pharmacology and Drug Research & Development, Semmelweis University, 1089 Budapest, Hungary

8 Institute of Clinical Pharmacy, University of Szeged, 6725 Szeged, Hungary

* Correspondence: csupor.dezso@szte.hu; Tel.: +(36-62) 544 944

FIGURE LEGENDS

Figure S1. Leave-One-Out Sensitivity Analysis on adenoma detection rate

Figure S2. Forest plot of proportion of no and mild peristalsis (PNMP) in colonoscopy and upper endoscopy

Figure S3. Leave-One-Out Sensitivity Analysis on the proportion of no peristalsis

Figure S4. Leave-One-Out Sensitivity Analysis on the ease of examination for the operator

Figure S5. Forest plot of withdrawal time in colonoscopy

Figure S6. Forest plot of adverse drug reactions

Figure S7. Risk of bias assessment of the analyzed studies (RoB2)

TABLE LEGENDS

Table S1. Versions of the search key in different databases

Table S2. Sponsor/Funding Source of the included studies

Table S3. Summary for quality of evidence, GRADE assessment

Table S4. Additional baseline characteristics of the RCTs with colonoscopy and upper endoscopy patients investigated in the systematic review and meta-analysis

Table S5. PRISMA 2020 Checklist

Table S1. Versions of the search key in different databases

**Searchkey used:**
**('mint' OR 'peppermint' OR 'Mentha' OR 'L-Menthol' OR 'menthol') AND ('endoscope' OR 'endoscopy' OR 'colonoscope' OR 'colonoscopy' OR 'gastroscope' OR 'gastroscopy' OR 'esophagogastroduodenoscopy' OR 'enteroscope' OR 'enteroscopy' OR 'duodenoscope' OR 'duodenoscopy' OR 'esophagoscope' OR 'esophagoscopy' OR 'endoscopic ultrasound' OR 'Endoscopic Retrograde Cholangiopancreatography' OR 'ERCP' OR 'adenoma detection rate' OR 'ADR')**
**Pubmed**<br>*Used the advanced search builder without restrictions.*
("mentha"[MeSH Terms] OR "mentha"[All Fields] OR "mint"[All Fields] OR ("mentha piperita"[MeSH Terms] OR ("mentha"[All Fields] AND "piperita"[All Fields]) OR "mentha piperita"[All Fields] OR "peppermint"[All Fields]) OR ("mentha"[MeSH Terms] OR "mentha"[All Fields] OR "menthae"[All Fields]) OR "L-Menthol"[All Fields] OR ("menthol"[MeSH Terms] OR "menthol"[All Fields] OR "menthol s"[All Fields] OR "mentholated"[All Fields] OR "mentholation"[All Fields] OR "menthols"[All Fields])) AND ("endoscope s"[All Fields] OR "endoscoped"[All Fields] OR "endoscopes"[MeSH Terms] OR "endoscopes"[All Fields] OR "endoscope"[All Fields] OR "endoscopical"[All Fields] OR "endoscopically"[All Fields] OR "endoscopy"[MeSH Terms] OR "endoscopy"[All Fields] OR "endoscopic"[All Fields] OR ("endoscopie"[All Fields] OR "endoscopy"[MeSH Terms] OR "endoscopy"[All Fields] OR "endoscopies"[All Fields] OR "endoscopy s"[All Fields]) OR ("colonoscope s"[All Fields] OR "colonoscoped"[All Fields] OR "colonoscopes"[MeSH Terms] OR "colonoscopes"[All Fields] OR "colonoscope"[All Fields] OR "colonoscopic"[All Fields] OR "colonoscopically"[All Fields]) OR ("colonoscopy"[MeSH Terms] OR "colonoscopy"[All Fields] OR "colonoscopies"[All Fields]) OR ("gastroscoped"[All Fields] OR "gastroscopes"[MeSH Terms] OR "gastroscopes"[All Fields] OR "gastroscope"[All Fields] OR "gastroscopic"[All Fields] OR "gastroscopically"[All Fields]) OR ("gastroscopy"[MeSH Terms] OR "gastroscopy"[All Fields] OR "gastroscopies"[All Fields]) OR ("endoscopy, digestive system"[MeSH Terms] OR ("endoscopy"[All Fields] AND "digestive"[All Fields] AND "system"[All Fields]) OR "digestive system endoscopy"[All Fields] OR "esophagogastroduodenoscopies"[All Fields] OR "oesophagogastroduodenoscopies"[All Fields] OR "oesophagogastroduodenoscopy"[All Fields] OR "esophagogastroduodenoscopy"[All Fields]) OR ("enteroscope"[All Fields] OR "enteroscopes"[All Fields] OR "enteroscopic"[All Fields] OR "enteroscopically"[All Fields]) OR ("enteroscopies"[All Fields] OR "enteroscopy"[All Fields]) OR ("duodenoscopes"[MeSH Terms] OR "duodenoscopes"[All Fields] OR "duodenoscope"[All Fields] OR "duodenoscopic"[All Fields]) OR ("duodenoscopy"[MeSH Terms] OR "duodenoscopy"[All Fields] OR "duodenoscopies"[All Fields]) OR ("esophagoscopes"[MeSH Terms] OR "esophagoscopes"[All Fields] OR "esophagoscopic"[All Fields] OR "oesophagoscope"[All Fields] OR "esophagoscope"[All Fields] OR "oesophagoscopic"[All Fields]) OR ("esophagoscopy" [MeSH Terms] OR "esophagoscopy" [All Fields] OR "esophagoscopies" [All Fields] OR "oesophagoscopies" [All Fields] OR "oesophagoscopy" [All Fields]) OR ("endosonography" [MeSH Terms] OR "endosonography" [All Fields] OR ("endoscopic" [All Fields] AND "ultrasound" [All Fields]) OR "endoscopic ultrasound" [All Fields]) OR ("cholangiopancreatography, endoscopic retrograde" [MeSH Terms] OR ("cholangiopancreatography" [All Fields] AND "endoscopic" [All Fields] AND "retrograde" [All Fields]) OR "endoscopic retrograde cholangiopancreatography" [All Fields] OR ("endoscopic" [All Fields] AND "retrograde" [All Fields] AND "cholangiopancreatography" [All Fields])) OR ("cholangiopancreatography, endoscopic retrograde" [MeSH Terms] OR ("cholangiopancreatography" [All Fields] AND "endoscopic" [All Fields] AND "retrograde" [All Fields]) OR "endoscopic retrograde cholangiopancreatography" [All Fields] OR "ercp" [All Fields]) OR (("adenoma" [MeSH Terms] OR "adenoma" [All Fields] OR "adenomas" [All Fields] OR "adenoma s" [All Fields]) AND ("detect" [All Fields] OR "detectabilities" [All Fields] OR "detectability" [All Fields] OR "detectable" [All Fields] OR "detectables" [All Fields] OR "detectably" [All Fields] OR "detected" [All Fields] OR "detectible" [All Fields] OR "detecting" [All Fields] OR "detection" [All Fields] OR "detections" [All Fields] OR "detects" [All Fields]) AND ("j rehabil assist technol eng" [Journal] OR "rate" [All Fields])) OR "ADR" [All Fields])
**EMBASE**<br>*Used the advanced search builder with no restrictions and unchecked mapping.*
(„mint”/exp OR mint OR „peppermint”/exp OR peppermint OR „mentha”/exp OR mentha OR „l menthol”/exp OR „l menthol” OR „menthol”/exp OR menthol) AND („endoscope”/exp OR endoscope OR „endoscopy”/exp OR endoscopy OR „colonoscope”/exp OR colonoscope OR „colonoscopy”/exp OR colonoscopy OR „gastroscope”/exp OR gastroscope OR „gastroscopy”/exp OR gastroscopy OR „esophagogastroduodenoscopy”/exp OR esophagogastroduodenoscopy OR „enteroscope”/exp OR enteroscope OR „enteroscopy”/exp OR enteroscopy OR „duodenoscope”/exp OR duodenoscope OR „duodenoscopy”/exp OR duodenoscopy OR „esophagoscope”/exp OR esophagoscope OR „esophagoscopy”/exp OR esophagoscopy OR „endoscopic ultrasound”/exp OR „endoscopic ultrasound” OR (endoscopic AND („ultrasound”/exp OR ultrasound)) OR „endoscopic retrograde cholangiopancreatography”/exp OR „endoscopic retrograde cholangiopancreatography” OR (endoscopic AND retrograde AND („cholangiopancreatography”/exp OR cholangiopancreatography)) OR „ercp”/exp OR ercp OR „adenoma detection rate”/exp OR „adenoma detection rate” OR ((„adenoma”/exp OR adenoma) AND („detection”/exp OR detection) AND rate) OR adr)
**Scopus**<br>*Used the advanced search builder without restrictions, did not use (’) marks in the search key.*
(mint OR peppermint OR mentha OR l-menthol OR menthol) AND (endoscope OR endoscopy OR colonoscope OR colonoscopy OR gastroscope OR gastroscopy OR esophagogastroduodenoscopy OR enteroscope OR enteroscopy OR duodenoscope OR duodenoscopy OR esophagoscope OR esophagoscopy OR endoscopic AND ultrasound OR endoscopic AND retrograde AND cholangiopancreatography OR ercp OR adenoma AND detection AND rate OR adr)
**Web of Science**<br>*Used the advanced search builder without restrictions.*
ALL(('mint' OR 'peppermint' OR 'Mentha' OR 'L-Menthol' OR 'menthol') AND ('endoscope' OR 'endoscopy' OR 'colonoscope' OR 'colonoscopy' OR 'gastroscope' OR 'gastroscopy' OR 'esophagogastroduodenoscopy' OR 'enteroscope' OR 'enteroscopy' OR 'duodenoscope' OR 'duodenoscopy' OR 'esophagoscope' OR 'esophagoscopy' OR 'endoscopic ultrasound' OR 'Endoscopic Retrograde Cholangiopancreatography' OR 'ERCP' OR 'adenoma detection rate' OR 'ADR'))
**Cochrane Library (TRIALS)**<br>*Used the advanced search within All text without restrictions.*
(('mint' OR 'peppermint' OR 'Mentha' OR 'L-Menthol' OR 'menthol') AND ('endoscope' OR 'endoscopy' OR 'colonoscope' OR 'colonoscopy' OR 'gastroscope' OR 'gastroscopy' OR 'esophagogastroduodenoscopy' OR 'enteroscope' OR 'enteroscopy' OR 'duodenoscope' OR 'duodenoscopy' OR 'esophagoscope' OR 'esophagoscopy' OR 'endoscopic ultrasound' OR 'Endoscopic Retrograde Cholangiopancreatography' OR 'ERCP' OR 'adenoma detection rate' OR 'ADR'))

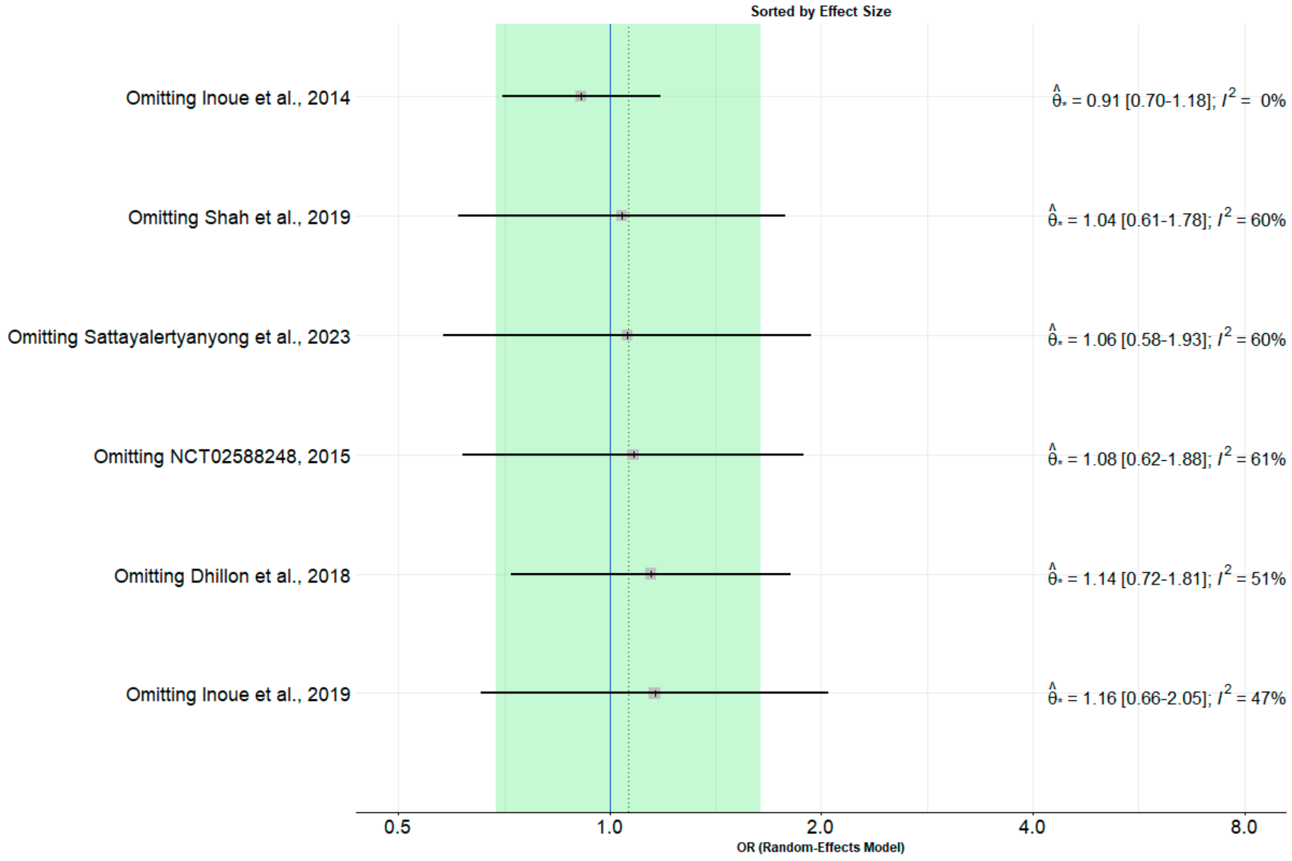

Figure S1. Leave-One-Out Sensitivity Analysis on adenoma detection rate

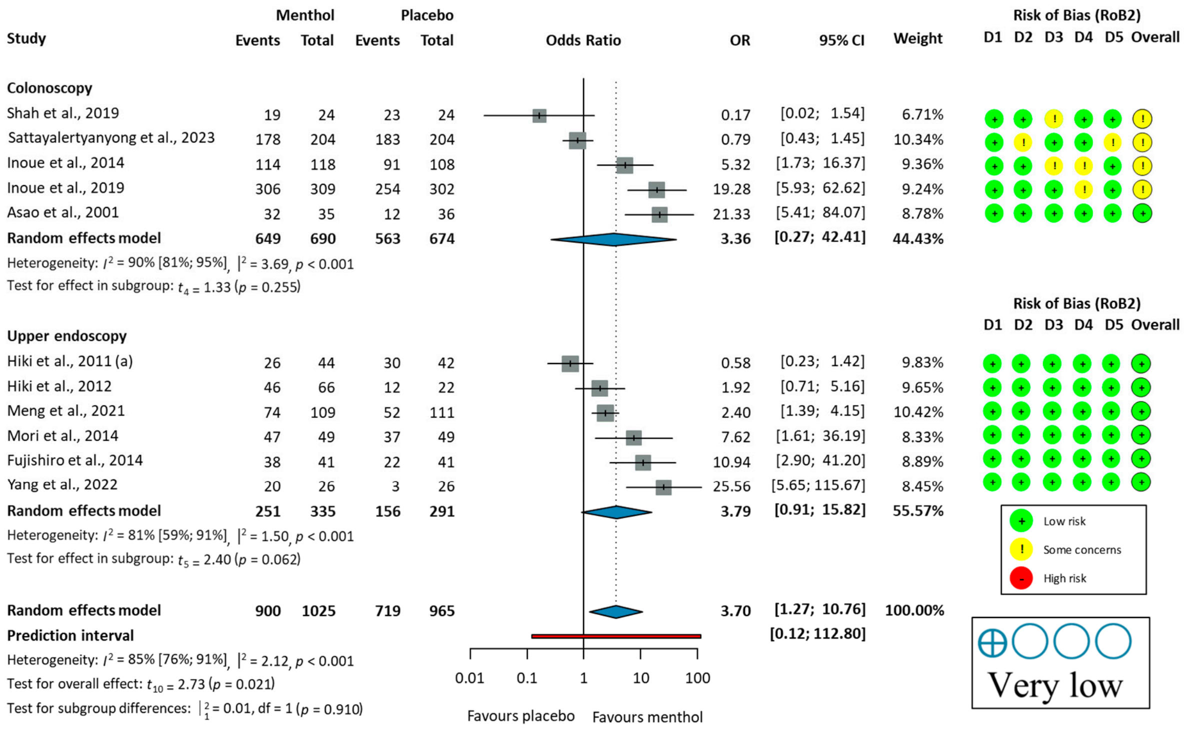

Figure S2. Forest plot of proportion of no and mild peristalsis (PNMP) in colonoscopy and upper endoscopy

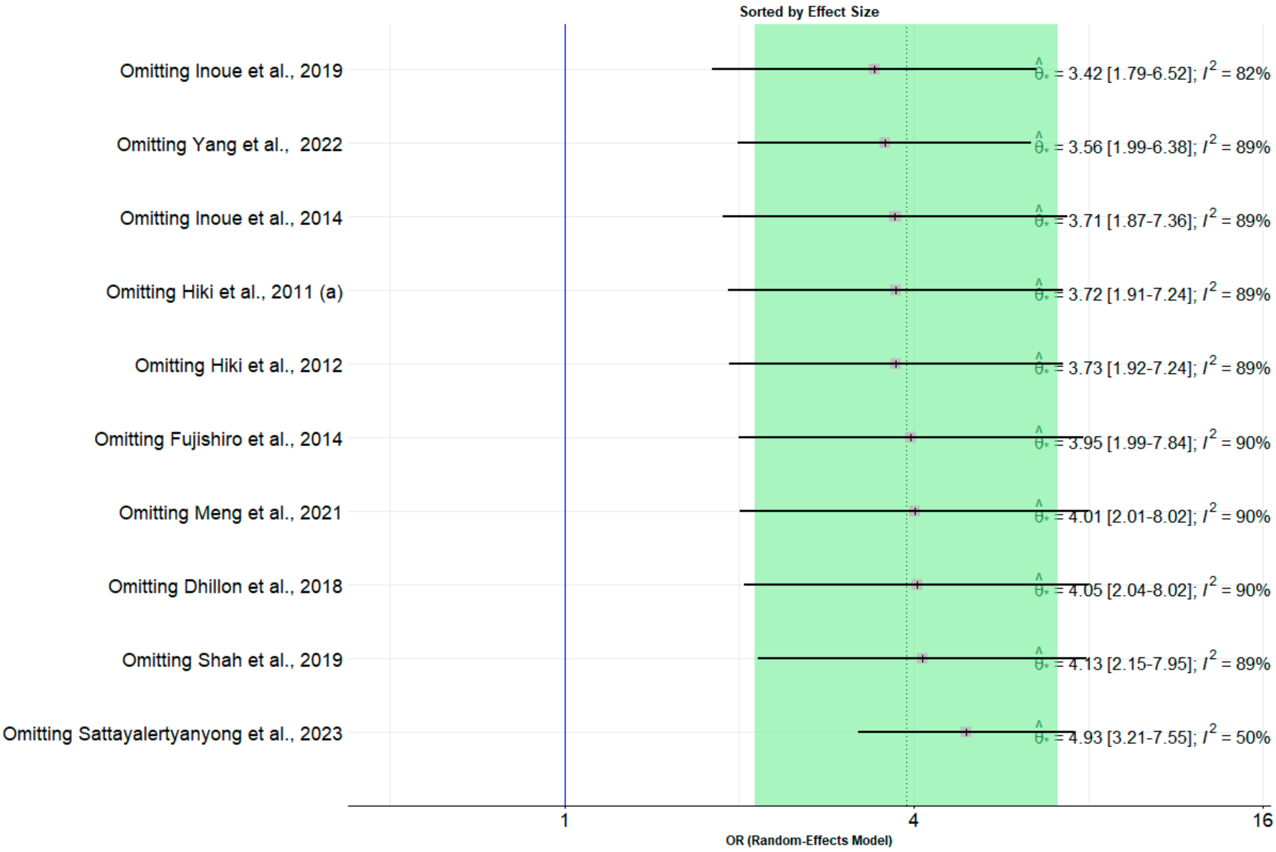

Figure S3. Leave-One-Out Sensitivity Analysis on the proportion of no peristalsis

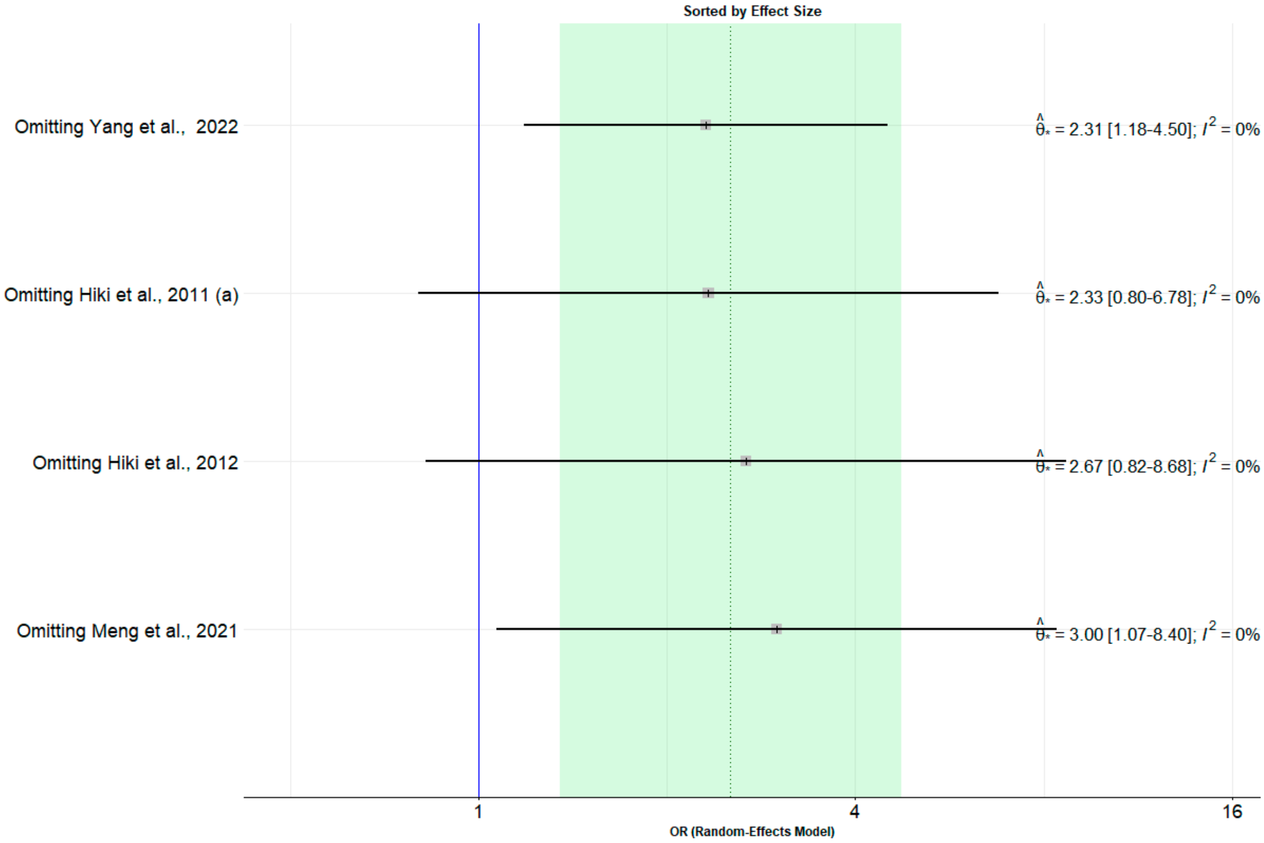

Figure S4. Leave-One-Out Sensitivity Analysis on the ease of examination for the operator

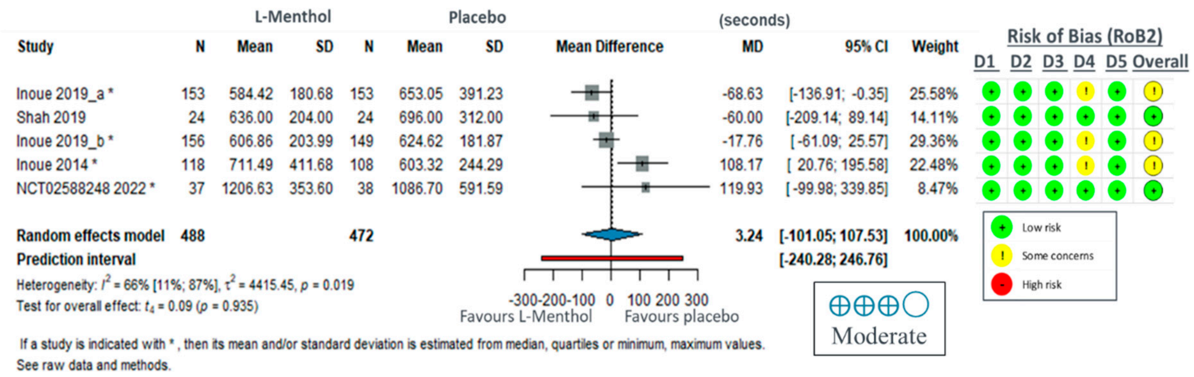

Figure S5. Forest plot of withdrawal time in colonoscopy

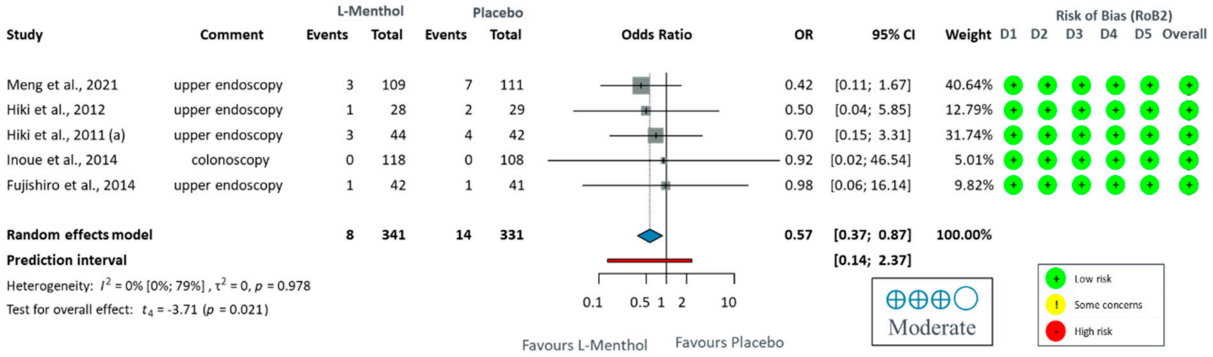

Figure S6. Forest plot of adverse drug reactions

Endoscopy type	Study ID	Experimental	Comparator	Outcome	D1	D2	D3	D4	D5	Overall
Colonoscopy	Dhillon_2018	L-Menthol	Placebo	Adenoma Detection Rate	+	!	+	+	!	!
Colonoscopy	Inoue_2014	L-Menthol	Placebo	Adenoma Detection Rate	+	+	+	!	+	!
Colonoscopy	Shah_2019	Peppermint oil	Placebo	Adenoma Detection Rate	+	!	+	+	+	!
Colonoscopy	Inoue_2019	L-Menthol	Placebo	Adenoma Detection Rate	+	+	+	!	+	!
Colonoscopy	NCT02588248_2015	L-Menthol	Placebo	Adenoma Detection Rate	+	+	+	+	+	+
Colonoscopy	Sattayalertyanyong_2023	Peppermint oil	Placebo	Adenoma Detection Rate	+	!	+	+	!	!
Colonoscopy	Asao_2001	Peppermint oil	Placebo	Adenoma Detection Rate	!	!	+	!	+	!
Colonoscopy	Han_2020	IBGard	Placebo	Adenoma Detection Rate	+	+	+	+	+	+

\+ Low risk
! Some concerns
\- High risk

D1 Randomisation process
D2 Deviations from the intended interver
D3 Missing outcome data
D4 Measurement of the outcome
D5 Selection of the reported result

Endoscopy type	Study ID	Experimental	Comparator	Outcome	D1	D2	D3	D4	D5	Overall
Colonoscopy	Dhillon_2018	L-Menthol	Placebo	Proportion of Peristalsis	+	!	+	+	!	!
Colonoscopy	Inoue_2014	L-Menthol	Placebo	Proportion of Peristalsis	+	+	!	!	+	!
Colonoscopy	Shah_2019	Peppermint oil	Placebo	Proportion of Peristalsis	+	+	!	+	+	!
Colonoscopy	Asao_2001	Peppermint oil	Placebo	Proportion of Peristalsis	-	!	!	-	-	-
Colonoscopy	Inoue_2019	L-Menthol	Placebo	Proportion of Peristalsis	+	+	+	!	+	!
Colonoscopy	Yoshida_2014	L-Menthol	Placebo	Proportion of Peristalsis	-	-	+	-	+	-
Colonoscopy	Sattayalertyanyong_2023	Peppermint oil	Placebo	Proportion of Peristalsis	+	!	+	+	!	!
Colonoscopy	Al Moussawi_2017	Colpermin	Placebo	Proportion of Peristalsis	+	+	+	+	+	+
Colonoscopy	Shavakhi_2012	Colpermin	Placebo	Proportion of Peristalsis	+	+	+	+	+	+

Endoscopy type	Study ID	Experimental	Comparator	Outcome	D1	D2	D3	D4	D5	Overall
Upper endoscopy	Fujishiro_2013	L-menthol	Placebo	Proportion of Peristalsis	+	+	+	+	+	+
Upper endoscopy	Hiki_2011a	L-Menthol	Placebo	Proportion of Peristalsis	+	+	+	+	+	+
Upper endoscopy	Hiki_2012	L-Menthol	Placebo	Proportion of Peristalsis	+	+	+	+	+	+
Upper endoscopy	Meng_2021	L-Menthol	Placebo	Proportion of Peristalsis	+	+	+	+	+	+
Upper endoscopy	Mori_2014	L-Menthol	Placebo	Proportion of Peristalsis	+	+	+	+	+	+
Upper endoscopy	Yang_2022	L-Menthol	Placebo	Proportion of Peristalsis	+	+	+	+	+	+

Endoscopy type	Study ID	Experimental	Comparator	Outcome	D1	D2	D3	D4	D5	Overall
Upper endoscopy	Hiki_2011a	L-Menthol	Placebo	Ease of Examination	+	+	+	+	+	+
Upper endoscopy	Hiki_2012	L-Menthol	Placebo	Ease of examination	+	+	+	+	+	+
Upper endoscopy	Meng_2021	L-Menthol	Placebo	Ease of Examination	+	+	+	+	+	+
Upper endoscopy	Yang_2022	L-Menthol	Placebo	Ease of Examination	+	+	+	+	+	+
Colonoscopy	Sattayalertyanyong_2023	Peppermint oil	Placebo	Ease of examination	+	!	+	+	!	!
Colonoscopy	Al Moussawi_2017	Colpermin	placebo	ease of examination	+	+	+	+	+	+
Colonoscopy	Han_2020	IBGard	placebo	ease of examination	+	+	+	+	+	+

Endoscopy type	Study ID	Experimental	Comparator	Outcome	D1	D2	D3	D4	D5	Overall
Colonoscopy	Inoue_2014	L-Menthol	Placebo	Withdrawal Time	+	+	+	!	+	!
Colonoscopy	Shah_2019	Peppermint oil	Placebo	Withdrawal Time	+	+	+	+	+	+
Colonoscopy	Inoue_2019	L-Menthol	Placebo	Withdrawal time	+	+	+	!	+	!
Colonoscopy	NCT02588248_2015	L-Menthol	Placebo	Withdrawal time	+	+	+	+	+	+
Colonoscopy	Sattayalertyanyong_2023	Peppermint oil	Placebo	Withdrawal time	+	!	+	+	!	!
Colonoscopy	Han_2020	IBGard	Placebo	Withdrawal time	+	+	+	+	+	+

Endoscopy type	Study ID	Experimental	Comparator	Outcome	D1	D2	D3	D4	D5	Overall
Colonoscopy	Asao_2001	Peppermint oil	Placebo	Adverse events	!	!	+	!	!	!
Colonoscopy	Yoshida_2014	L-Menthol	Placebo	Adverse events	!	!	+	!	!	!
Colonoscopy	Sattayalertyanyong_2023	Peppermint oil	Placebo	Adverse events	+	!	+	+	!	!
Colonoscopy	NCT02588248_2015	L-Menthol	Placebo	Adverse events	+	+	+	+	+	+
Colonoscopy	Inoue_2014	L-Menthol	Placebo	Adverse events	+	+	+	!	+	!
Colonoscopy	Dhillon_2018	L-Menthol	Placebo	Adverse events	+	!	+	+	!	!
Colonoscopy	Inoue_2019	L-Menthol	Placebo	Adverse events	+	+	+	!	+	!
Colonoscopy	Shah_2019	Peppermint oil	Placebo	Adverse events	+	+	+	+	+	+
Upper endoscopy	Imagawa_2012	Peppermint oil	Placebo	Adverse events	+	+	+	+	+	+
Upper endoscopy	Hiki_2011a	L-Menthol	Placebo	Adverse events	+	+	+	+	+	+
Upper endoscopy	Meng_2021	L-Menthol	Placebo	Adverse events	+	+	+	+	+	+
Upper endoscopy	Mori_2014	L-Menthol	Placebo	Adverse events	+	+	+	+	+	+
Upper endoscopy	Fujishiro_2013	L-menthol	Placebo	Adverse events	+	+	+	+	+	+
Upper endoscopy	Yang_2022	L-Menthol	Placebo	Adverse events	+	+	+	+	+	+
Upper endoscopy	Hiki_2011b	L-Menthol	Placebo	Adverse events	+	+	+	+	+	+
Upper endoscopy	Hiki_2012	L-Menthol	Placebo	Adverse events	+	+	+	+	+	+

Figure S7. Risk of bias assessment of the analyzed studies (RoB2)

Table S2. Sponsor/Funding Source of the included studies

Study (First Author, Year)	Procedure	Sponsor/Funding Source
Al Moussawi et al., 2017 [65]	Colonoscopy	Not reported
Asao et al., 2001 [30]	Colonoscopy	Not reported
Dhillon et al., 2018 [51]	Colonoscopy	None
Fujishiro et al., 2014 [53]	Upper Endoscopy	Nihon Pharmaceutical Co., Ltd.
Han et al., 2021 [66]	Colonoscopy	Academic
Hiki et al., 2011a [58]	Upper Endoscopy	Nihon Pharmaceutical Co., Ltd.
Hiki et al., 2011b [59]	Upper Endoscopy	Nihon Pharmaceutical Co., Ltd.
Hiki et al., 2012 [73]	Upper Endoscopy	Nihon Pharmaceutical Co., Ltd.
Imagawa et al., 2012 [60]	Upper Endoscopy	Not reported
Inoue et al., 2014 [54]	Colonoscopy	Hospital-funded
Inoue et al., 2020 [55]	Colonoscopy	Not reported
Meng et al., 2021 [61]	Upper Endoscopy	Not reported
Mori et al., 2014 [62]	Upper Endoscopy	Not reported
NCT02588248, 2015 [52]	Colonoscopy	Academic/hospital-funded
Sattayalertyanyong et al., 2023 [56]	Colonoscopy	None
Shah et al., 2019 [57]	Colonoscopy	Hospital-funded
Shavakhi et al., 2012 [67]	Colonoscopy	Academic funded
Yang et al., 2022 [63]	Upper Endoscopy	Not reported
Yoshida et al., 2014 [64]	Colonoscopy	Not reported

Table S3. Summary for quality of evidence, GRADE assessment [35]

Certainty assessment							Summary of findings				
Participants (studies) Follow-up	Risk of bias	Inconsistency	Indirectness	Imprecision	Publication bias	Overall certainty of evidence	Study event rates (%)		Relative effect (95% CI)	Anticipated absolute effects	
							With placebo	With L-menthol		Risk with placebo	Risk difference with L-menthol
**Quality of endoscopy in colonoscopy (assessed with: Adenoma detection rate)**											
1490 (6 RCTs)	serious[a]	not serious	serious[b]	serious[c]	none	⨁◯◯◯ Very low[a,b,c]	338/737 (45.9%)	351/753 (46.6%)	**OR 1.06** (0.69 to 1.64)	338/737 (45.9%)	**14 more per 1000** (from 90 fewer to 123 more)
**Proportion of no peristalsis in colonoscopy + upper endoscopy**											
1943 (10 RCTs)	serious[d,e]	not serious	serious[f]	not serious	none	⨁◯◯◯ Very low[d,e,f]	253/941 (26.9%)	560/1002 (55.9%)	**OR 3.88** (2.13 to 7.07)	253/941 (26.9%)	**319 more per 1000** (from 170 more to 453 more)
**Proportion of no or mild peristalsis in colonoscopy + upper endoscopy**											
1990 (11 RCTs)	serious[e,g]	not serious	serious[h]	not serious	none	⨁◯◯◯ Very low[f,g,h]	719/965 (74.5%)	900/1025 (87.8%)	**OR 3.70** (1.27 to 10.76)	719/965 (74.5%)	**170 more per 1000** (from 43 more to 224 more)
**Proportion of no peristalsis in upper endoscopy (after application of L-menthol)**											
626 (6 RCTs)	serious[e]	not serious	not serious	not serious	none	⨁⨁⨁◯ Moderate[f]	156/291 (53.6%)	251/335 (74.9%)	**OR 3.79** (0.91 to 15.82)	156/291 (53.6%)	**278 more per 1000** (from 24 fewer to 412 more)

Certainty assessment							Summary of findings				
Participants (studies) Follow-up	Risk of bias	Inconsistency	Indirectness	Imprecision	Publication bias	Overall certainty of evidence	Study event rates (%)		Relative effect (95% CI)	Anticipated absolute effects	
							With placebo	With L-menthol		Risk with placebo	Risk difference with L-menthol
**Proportion of no peristalsis in upper endoscopy (at the end of endoscopy)**											
516 (5 RCTs)	serious[e]	not serious	not serious	not serious	none	⨁⨁⨁◯ Moderate[f]	113/236 (47.9%)	227/280 (81.1%)	**OR 6.17** (2.00 to 18.99)	113/236 (47.9%)	**371 more per 1000** (from 169 more to 467 more)
**Ease of examination for operator in upper endoscopy**											
464 (4 RCTs)	serious[e]	not serious	not serious	not serious	none	⨁⨁⨁◯ Moderate[f]	140/208 (67.3%)	206/256 (80.5%)	**OR 2.53** (1.35 to 4.73)	140/208 (67.3%)	**166 more per 1000** (from 62 more to 234 more)
**Total adverse events in colonoscopy + upper endoscopy**											
4449 (16 RCTs)	not serious	serious[i]	not serious	not serious	none	⨁⨁⨁◯ Moderate[i]	77/1111 (6.9%)	91/3338 (2.7%)	**OR 0.93** (0.69 to 1.24)	77/1111 (6.9%)	**5 fewer per 1000** (from 20 fewer to 15 more)
**Total adverse drug reactions in colonoscopy + upper endoscopy**											
672 (5 RCTs)	not serious	serious[i]	not serious	not serious	none	⨁⨁⨁◯ Moderate[i]	14/331 (4.2%)	8/341 (2.3%)	**OR 0.57** (0.37 to 0.87)	14/331 (4.2%)	**18 fewer per 1000** (from 26 fewer to 5 fewer)
**Withdrawal time (assessed with: seconds)**											
1368 (5 RCTs)	serious[j]	not serious	not serious	not serious	none	⨁⨁⨁◯ Moderate[j]	676	692	-	676	MD **0.7 seconds higher** (69.54 lower to 70.94 higher)

Explanations

a. Downgraded once due to concerns in RoB2 Domains 2 (deviations from interventions), 4 (outcome measurement), and 5 (selective reporting) in 5/6 studies. These issues introduce uncertainty in the estimated effect.

b. Downgraded once due to heterogeneity in the clinical methods (screening vs. follow-up colonoscopies), limiting generalizability.

c. Downgraded once because confidence intervals crossed the null effect in 5/6 studies, indicating uncertainty about benefit or harm.

d. Downgraded once for colonoscopy studies (concerns in RoB2 Domains 2, 3, 4, 5). Upper endoscopy studies had low risk.

e. Downgraded once due to unblinded outcome assessment (grading variability among endoscopists), affecting outcome measurement (RoB2 Domain 4).

f. Downgraded once for colonoscopy studies due to mixed populations (screening vs. follow-up). Upper endoscopy studies had homogeneous indications.

g. Downgraded once for colonoscopy studies (concerns in RoB2 Domains 2, 3, 4, 5). Upper endoscopy studies had a low risk.

h. Downgraded once for colonoscopy studies due to mixed populations (screening vs. follow-up). Upper endoscopy studies had homogeneous indications.

i. Downgraded once due to heterogeneity in adverse event measurement methods (e.g., patient-reported vs. observed), leading to unexplained variability.

j. Downgraded once for RoB2 Domain 4 (lack of blinding for colonoscopists), introducing performance bias.

Table S4. Additional baseline characteristics of the RCTs with colonoscopy and upper endoscopy patients investigated in the systematic review and meta-analysis

Study (Year)	Sample Size	Centers	Procedure Type	Population Age (Mean)	Gender female %	Sedation Type	Endoscopist Experience	Comorbidities
Asao et al., 2001 (30)	I: 409<br>C: 36	unicentric	Colonoscopy	mean, SD, range<br>I: 56.8+/-11.3 (25-89)<br>C: 55.2+/-11.9 (21-76)	I: 24.45%<br>C: 27.78%	not mentioned	not mentioned	Irritable bowel syndrome, Cardiovascular, Hypertension, Prostate hypertrophy, Asthma, Diabetes, Glaucoma
Dhillon et al., 2018 (51)	I: 61<br>C: 61	unicentric	Colonoscopy	not mentioned	not mentioned	not mentioned	not mentioned	not mentioned
Fujishiro et al., 2014 (53)	I: 41<br>C: 41	multicentric	Upper endoscopy	mean, range<br>I: 70.4 (58-88)<br>C: 69.6 (48-82)	I: 19.51%<br>C: 19.51%	benzodiazepine and pethidine hydrochloride	Board-certified endoscopists	not mentioned
Hiki et al., 2011a (58)	I: 45<br>C: 42	multicentric	Upper endoscopy	mean, range<br>I: 64.5 (28-85)<br>C: 62.4 (28-84)	I: 40%<br>C: 35.7%	unsedated	not mentioned	not mentioned
Hiki et al., 2011b (59)	I (80mg): 6<br>I (160mg): 6<br>I (320mg): 6<br>C: 6	multicentric	Upper endoscopy	mean, range<br>I (80mg): 48.2 (36-62)<br>I (160mg): 46.7 (39-57)<br>I (320mg) 47.0 (35-64)<br>C: 51.2 (41-64)	I: 0%<br>C: 0%	unsedated	not mentioned	not mentioned
Hiki et al., 2012 (73)	I (0.4%): 30<br>I (0.8%): 28<br>I: (1.6%) 29<br>C: 29	multicentric	Upper endoscopy	mean, range<br>I (0.4%): 58.4 (22-76)<br>I (0.8%): 57.8 (29-81)<br>I: (1.6%) 62.7 (40-82)<br>C: 57.0 (22-77)	I: 43.3%<br>C: 37.9%	not mentioned	not mentioned	not mentioned
Imagawa et al., 2012 (60)	I: 1893<br>C: 156	multicentric	Upper endoscopy	mean, SD<br>I: 73+/-11<br>C: 73+/-11	I: 36.6%<br>C: 35.9%	not mentioned	more than three years experience	not mentioned
Inoue et al., 2014 (54)	I: 118<br>C: 108	unicentric	Colonoscopy	median, range<br>I: 68 (33-87)<br>C: 66 (27-90)	I: 44.9%<br>C: 50%	Midazolam or pentazocine (on request of patient)	previously performed over 1000 colonoscopies	Prostatic hyperplasia, Glaucoma, Cardiac disease, Arrhythmia

Inoue et al., 2020 (55)	I: 153<br>C: 153	unicentric	Colonoscopy	median, range<br>I: 62 (32-89)<br>C: 60 (29-84)	I: 51%<br>C: 52.3%	not mentioned	previously performed over 1000 colonoscopies, and trainees	Prostatic hyperplasia, Glaucoma,<br>Cardiac disease, Arrhythmia, Diabetes, Hypertension, Dyslipidemia
Meng et al., 2021 (61)	I: 109<br>C: 111	multicentric	Upper endoscopy	mean, SD<br>I: 51.64+/-12.84<br>C: 51.44+/-13.67	I: 53.21%<br>C: 44.14%	unsedated	experienced professionals	not mentioned
Mori et al., 2014 (62)	I: 49<br>C: 49	unicentric	Upper endoscopy	mean, range<br>I: 64 (25-94)<br>C: 59 (26-82)	I: 53.1%<br>C: 40.8%	unsedated	not mentioned	not mentioned
NCT02588248, 2015 (52)	I: 37<br>C: 38	unicentric	Colonoscopy	median, SD<br>I: 60.5+/-7.77<br>C: 67.73+/-8.8	I: 67.6%<br>C: 55.3%	conscious sedation	not mentioned	not mentioned
Sattayalertyanyong et al., 2023 (56)	I: 204<br>C: 204	unicentric	Colonoscopy	mean, SD<br>I: 60.4+/-10.2<br>C: 59.6+/-10.2	I: 68.6%<br>C: 65.7%	propofol, fentanyl, midazolam, lidocaine	not mentioned	Hypertension, Dyslipidemia, Diabetes mellitus, Chronic kidney disease, Irritable bowel syndrome
Shah et al., 2019 (57)	I: 24<br>C: 24	unicentric	Colonoscopy	mean, SD<br>I: 53.8+/-3.9<br>C: 54.8+/-4.9	I: 45.8%<br>C: 79.2%	conscious sedation	skilled endoscopists	Diabetes mellitus
Yang et al., 2022 (63)	I: 26<br>C: 26	unicentric	Upper endoscopy	mean, SD<br>I: 81.7+/-7.8<br>C: 82.6+/-5.3	I: 30.8%<br>C: 34.6%	unsedated	experienced endoscopists	Prostatic hyperplasia, Cardiac disease, Glaucoma
Yoshida et al., 2014 (64)	I: 65<br>C: 27	unicentric	Colonoscopy	mean, SD<br>I: 71.7+/-9.6<br>C: 70.9+/-8	I: 24.6%<br>C: 33.3%	not mentioned	expert endoscopist	not mentioned

Table S5. PRISMA 2020 Checklist [25]

Section and Topic	Item #	Checklist item	Location where item is reported
**TITLE**			
Title	1	Identify the report as a systematic review.	row 1
**ABSTRACT**			
Abstract	2	See the PRISMA 2020 for Abstracts checklist.	row 16
**INTRODUCTION**			
Rationale	3	Describe the rationale for the review in the context of existing knowledge.	1
Objectives	4	Provide an explicit statement of the objective(s) or question(s) the review addresses.	1
**METHODS**			
Eligibility criteria	5	Specify the inclusion and exclusion criteria for the review and how studies were grouped for the syntheses.	2.1, 2.4
Information sources	6	Specify all databases, registers, websites, organisations, reference lists and other sources searched or consulted to identify studies. Specify the date when each source was last searched or consulted.	2.2
Search strategy	7	Present the full search strategies for all databases, registers and websites, including any filters and limits used.	2.3
Selection process	8	Specify the methods used to decide whether a study met the inclusion criteria of the review, including how many reviewers screened each record and each report retrieved, whether they worked independently, and if applicable, details of automation tools used in the process.	2.4
Data collection process	9	Specify the methods used to collect data from reports, including how many reviewers collected data from each report, whether they worked independently, any processes for obtaining or confirming data from study investigators, and if applicable, details of automation tools used in the process.	2.5
Data items	10a	List and define all outcomes for which data were sought. Specify whether all results that were compatible with each outcome domain in each study were sought (e.g. for all measures, time points, analyses), and if not, the methods used to decide which results to collect.	2.6
	10b	List and define all other variables for which data were sought (e.g. participant and intervention characteristics, funding sources). Describe any assumptions made about any missing or unclear information.	2.6
Study risk of bias assessment	11	Specify the methods used to assess risk of bias in the included studies, including details of the tool(s) used, how many reviewers assessed each study and whether they worked independently, and if applicable, details of automation tools used in the process.	2.7
Effect measures	12	Specify for each outcome the effect measure(s) (e.g. risk ratio, mean difference) used in the synthesis or presentation of results.	2.9
Synthesis methods	13a	Describe the processes used to decide which studies were eligible for each synthesis (e.g. tabulating the study intervention characteristics and comparing against the planned groups for each synthesis (item #5)).	N/A
	13b	Describe any methods required to prepare the data for presentation or synthesis, such as handling of missing summary statistics, or data conversions.	2.9
	13c	Describe any methods used to tabulate or visually display results of individual studies and syntheses.	N/A
	13d	Describe any methods used to synthesize results and provide a rationale for the choice(s). If meta-analysis was performed, describe the model(s), method(s) to identify the presence and extent of statistical heterogeneity, and software package(s) used.	2.9
	13e	Describe any methods used to explore possible causes of heterogeneity among study results (e.g. subgroup analysis, meta-regression).	2.9
	13f	Describe any sensitivity analyses conducted to assess robustness of the synthesized results.	N/A
Reporting bias assessment	14	Describe any methods used to assess risk of bias due to missing results in a synthesis (arising from reporting biases).	2.9
Certainty assessment	15	Describe any methods used to assess certainty (or confidence) in the body of evidence for an outcome.	2.8

Section and Topic	Item #	Checklist item	Location where item is reported
**RESULTS**			
Study selection	16a	Describe the results of the search and selection process, from the number of records identified in the search to the number of studies included in the review, ideally using a flow diagram.	3.1
	16b	Cite studies that might appear to meet the inclusion criteria, but which were excluded, and explain why they were excluded.	Figure 1
Study characteristics	17	Cite each included study and present its characteristics.	3.2, Table 1
Risk of bias in studies	18	Present assessments of risk of bias for each included study.	3.5., Suppl. Figure S4
Results of individual studies	19	For all outcomes, present, for each study: (a) summary statistics for each group (where appropriate) and (b) an effect estimate and its precision (e.g. confidence/credible interval), ideally using structured tables or plots.	3.3
Results of syntheses	20a	For each synthesis, briefly summarise the characteristics and risk of bias among contributing studies.	3.3
	20b	Present results of all statistical syntheses conducted. If meta-analysis was done, present for each the summary estimate and its precision (e.g. confidence/credible interval) and measures of statistical heterogeneity. If comparing groups, describe the direction of the effect.	3.3
	20c	Present results of all investigations of possible causes of heterogeneity among study results.	3.3, 3.6
	20d	Present results of all sensitivity analyses conducted to assess the robustness of the synthesized results.	N/A
Reporting biases	21	Present assessments of risk of bias due to missing results (arising from reporting biases) for each synthesis assessed.	Suppl. Figure S4
Certainty of evidence	22	Present assessments of certainty (or confidence) in the body of evidence for each outcome assessed.	Suppl. Table S3
**DISCUSSION**			
Discussion	23a	Provide a general interpretation of the results in the context of other evidence.	4.
	23b	Discuss any limitations of the evidence included in the review.	4.1
	23c	Discuss any limitations of the review processes used.	4.1
	23d	Discuss implications of the results for practice, policy, and future research.	4.2
**OTHER INFORMATION**			
Registration and protocol	24a	Provide registration information for the review, including register name and registration number, or state that the review was not registered.	2
	24b	Indicate where the review protocol can be accessed, or state that a protocol was not prepared.	2
	24c	Describe and explain any amendments to information provided at registration or in the protocol.	N/A
Support	25	Describe sources of financial or non-financial support for the review, and the role of the funders or sponsors in the review.	row 531
Competing interests	26	Declare any competing interests of review authors.	row 548
Availability of data, code and other materials	27	Report which of the following are publicly available and where they can be found: template data collection forms; data extracted from included studies; data used for all analyses; analytic code; any other materials used in the review.	row 539

From: Page MJ, McKenzie JE, Bossuyt PM, Boutron I, Hoffmann TC, Mulrow CD, et al. The PRISMA 2020 statement: an updated guideline for reporting systematic reviews. BMJ 2021;372:n71. doi: 10.1136/bmj.n71. This work is licensed under CC BY 4.0. To view a copy of this license, visit https://creativecommons.org/licenses/by/4.0/ [25]

References

25. Page, M.J.; McKenzie, J.E.; Bossuyt, P.M.; Boutron, I.; Hoffmann, T.C.; Mulrow, C.D.; Shamseer, L.; Tetzlaff, J.M.; Akl, E.A.; Brennan, S.E.; et al. The PRISMA 2020 statement: An updated guideline for reporting systematic reviews. *BMJ* **2021**, *372*, n71.

30. Asao, T.; Mochiki, E.; Suzuki, H.; Nakamura , J.; Hirayama, I.; Morinaga, N.; Shoji, H.; Shitara, Y.; & Kuwano, H. An easy method for the intraluminal administration of peppermint oil before colonoscopy and its effectiveness in reducing colonic spasm. *Gastrointest. Endosc.* **2001**, *53*, 172–177.

35. GRADEpro GDT. GRADEpro Guideline Development Tool. McMaster University and Evidence Prime, 2023. Available online: https://www.gradepro.org/ (accessed on 18 January 2025).

51. Dhillon, A.S.; Alshankiti, S.; Khorasani-zadeh, A.; Sultanian, R.; Sandha, G.S.; Kohansal, A.R.; Montano-Loza, A.J.; Zepeda-Gomez, S. A247 L-menthol during colonoscopy for adenoma detection in an intermediate risk patient population: A double-blind, randomized controlled trial. J. Can. Assoc. Gastroenterol. 2018, 1, 360–361.

52. University Hospitals Cleveland Medical Center L-Menthol Injection as a Novel Technique During Colonoscopy (MINT-C) NCT02588248, 2015. Available online: https://clinicaltrials.gov/study/NCT02588248 (accessed on 18 June 2023).

53. Fujishiro, M.; Kaminishi, M.; Hiki, N.; Oda, I.; Fujisaki, J.; Uedo, N.; Kaise, M.; Tanabe, S.; Iguchi, M.; Matsuhashi, N.; et al. Efficacy of spraying l-menthol solution during endoscopic treatment of early gastric cancer: A phase III, multicenter, ran-domized, double-blind, placebo-controlled study. J. Gastroenterol. 2014, 49, 446–454.

54. Inoue, K.; Dohi, O.; Gen, Y.; Jo, M.; Mazaki, T.; Tokita, K.; Yoshida, N.; Okayama, T.; Kamada, K.; Katada, K.; et al. L-menthol improves adenoma detection rate during colonoscopy: A randomized trial. Endoscopy 2014, 46, 196–202.

55. Inoue, K.; Okuda, T.; Oka, K.; Sugino, S.; Endo, Y.; Ota, T.; Minagawa, Y.; Yasue, C.; Tsuji, T.; Katayama, T.; et al. Effects of L-Menthol and Carbon Dioxide on the Adenoma Detection Rate during Colonoscopy: L-Menthol and Carbon Dioxide on Colonoscopy. Digestion 2020, 101, 323–331.

56. Sattayalertyanyong, O.; Sathirawich, P.; Maipang, K.; Chukaewrungroj, P.; Limsrivilai, J.; Kaosombatwattana, U. Efficacy and safety of intraluminal peppermint oil during colonoscopy on colonic peristalsis and adenoma detection rate: A random-ized, double-blinded, placebo-controlled trial. Gastrointest. Endosc. 2023, 97, AB696–AB697.

57. Shah, I.; Baffy, N.J.; Horsley-Silva, J.L.; Langlais, B.T.; Ruff, K.C. Peppermint Oil to Improve Visualization in Screening Co-lonoscopy: A Randomized Controlled Clinical Trial. Gastroenterol. Res. 2019, 12, 141–147.

58. Hiki, N.; Kaminishi, M.; Yasuda, K.; Uedo, N.; Honjo, H.; Matsuhashi, N.; Hiratsuka, T.; Sekine, C.; Nomura, S.; Yahagi, N.; et al. Antiperistaltic effect and safety of L-menthol sprayed on the gastric mucosa for upper GI endoscopy: A phase III, mul-ticenter, randomized, double-blind, placebo-controlled study. Gastrointest. Endosc. 2011, 73, 932–941.

59. Hiki, N.; Kaminishi, M.; Yasuda, K.; Uedo, N.; Kobari, M.; Sakai, T.; Hiratsuka, T.; Ohno, K.; Honjo, H.; Nomura, S.; et al. Multicenter phase II randomized study evaluating dose-response of antiperistaltic effect of L-menthol sprayed onto the gas-tric mucosa for upper gastrointestinal endoscopy. Dig. Endosc. 2012, 24, 79–86.

60. Imagawa, A.; Hata, H.; Nakatsu, M.; Yoshida, Y.; Takeuchi, K.; Inokuchi, T.; Imada, T.; Kohno, Y.; Takahara, M.; Matsumo-to, K.; et al. Peppermint oil solution is useful as an antispasmodic drug for esophagogastroduodenoscopy, especially for el-derly patients. Dig. Dis. Sci. 2012, 57, 2379–2384.

61. Meng, F.; Li, W.; Zhi, F.; Li, Z.; Xue, Z.; He, S.; Chen, W.; Chen, Y.; Xing, X.; Yao, C.; et al. Antiperistaltic effect and safety of l-menthol oral solution on gastric mucosa for upper gastrointestinal endoscopy in Chinese patients: Phase III, multicenter, randomized, double-blind, placebo-controlled study. Dig. Endosc. 2021, 33, 1110–1119.

62. Mori, A.; Hachiya, H.; Yumura, T.; Ito, S.; Hayashi, S.; Nozaki, M.; Yoshida, A.; & Ohashi, N. l-Menthol sprayed on gastric mucosa causes edematous change. Endosc. Int. Open. 2014, 2, E51–E57.

63. Yang, T.C.; Chen, P.H.; Hou, M.C.; Peng, L.N.; Lin, M.H.; Chen, L.K.; & Huang, Y.H. Antiperistaltic effect and safety of L-menthol for esophagogastroduodenoscopy in the elderly with contraindication to hyoscine-N-butylbromide. Sci. Rep. 2022, 12, 10418.

64. Yoshida, N.; Naito, Y.; Hirose, R.; Ogiso, K.; Inada, Y.; Fernandopulle, N.; Kamada, K.; Katada, K.; Uchiyama, K.; Handa, O.; et al. Prevention of colonic spasm using L-menthol in colonoscopic examination. Int. J. Color. Dis. 2014, 29, 579–583.

65. Al Moussawi, H.; Al Khatib, M.; El Ahmar, M.; Al Masri, H.; Leddy, A.; Akel, T.; & Khalil, A. The effect of premedication with peppermint oil capsules (Colpermin) prior to colonoscopy: A double blind randomized placebo-controlled trial. Arab. J. Gastroenterol. 2017, 18, 220–223.

66. Han, J.Y.; Moosvi, Z.; Duh, E.; Park, S.; Albers, G.C.; Samarasena, J.B.; & Karnes, W. Oral IBGard™ Before Colonoscopy: A Single-Center Double-Blinded, Randomized, Placebo-Controlled Trial. Dig. Dis. Sci. 2021, 66, 1611–1619.

67. Shavakhi, A.; Ardestani, S.K.; Taki, M.; Goli, M.; Keshteli, A.H. Premedication with peppermint oil capsules in colonoscopy: A double blind placebo-controlled randomized trial study. Acta Gastroenterol. Belg. 2012, 75, 349–353.

73. Hiki, N.; Kaminishi, M.; Hasunuma, T.; Nakamura, M.; Nomura, S.; Yahagi, N.; Tajiri, H.; & Suzuki, H. A phase I study evaluating tolerability, pharmacokinetics, and preliminary efficacy of L-menthol in upper gastrointestinal endoscopy. Clin.

Pharmacol. Ther. 2011, 90, 221–228.
